# Impact of Enhanced Health Interventions for United States–Bound Refugees: Evaluating Best Practices in Migration Health

**DOI:** 10.4269/ajtmh.17-0725

**Published:** 2017-12-18

**Authors:** Tarissa Mitchell, Deborah Lee, Michelle Weinberg, Christina Phares, Nicola James, Kittisak Amornpaisarnloet, Lalita Aumpipat, Gretchen Cooley, Anita Davies, Valerie Daw Tin Shwe, Vasil Gajdadziev, Olga Gorbacheva, Chutharat Khwan-Niam, Alexander Klosovsky, Waritorn Madilokkowit, Diana Martin, Naing Zaw Htun Myint, Thi Ngoc Yen Nguyen, Thomas B. Nutman, Elise M. O’Connell, Luis Ortega, Sugunya Prayadsab, Chetdanai Srimanee, Wasant Supakunatom, Vattanachai Vesessmith, William M. Stauffer

**Affiliations:** 1 Division of Global Migration and Quarantine, Centers for Disease Control and Prevention, Atlanta, Georgia;; 2 International Organization for Migration, Bangkok, Thailand;; 3 Division of Parasitic Diseases and Malaria, Centers for Disease Control and Prevention, Atlanta, Georgia;; 4 International Organization for Migration, Washington, DC;; 5 Laboratory of Parasitic Diseases, National Institutes of Health, Bethesda, Maryland;; 6 Department of Medicine, University of Minnesota Medical School, Minneapolis, Minnesota

## Abstract

With an unprecedented number of displaced persons worldwide, strategies for improving the health of migrating populations are critical. United States–bound refugees undergo a required overseas medical examination to identify inadmissible conditions (e.g., tuberculosis) 2–6 months before resettlement, but it is limited in scope and may miss important, preventable infectious, chronic, or nutritional causes of morbidity. We sought to evaluate the feasibility and health impact of diagnosis and management of such conditions before travel. We offered voluntary testing for intestinal parasites, anemia, and hepatitis B virus infection, to U.S.-bound refugees from three Thailand–Burma border camps. Treatment and preventive measures (e.g., anemia and parasite treatment, vaccination) were initiated before resettlement. United States refugee health partners received overseas results and provided post-arrival medical examination findings. During July 9, 2012 to November 29, 2013, 2,004 refugees aged 0.5–89 years enrolled. Among 463 participants screened for seven intestinal parasites overseas and after arrival, helminthic infections decreased from 67% to 12%. Among 118 with positive *Strongyloides*-specific antibody responses, the median fluorescent intensity decreased by an average of 81% after treatment. The prevalence of moderate-to-severe anemia (hemoglobin < 10 g/dL) was halved from 14% at baseline to 7% at departure (McNemar *P* = 0.001). All 191 (10%) hepatitis B–infected participants received counseling and evaluation; uninfected participants were offered vaccination. This evaluation demonstrates that targeted screening, treatment, and prevention services can be conducted during the migration process to improve the health of refugees before resettlement. With more than 250 million migrants globally, this model may offer insights into healthier migration strategies.

## INTRODUCTION

Worldwide, an unprecedented 65 million people—approximately 1% of the world’s population—are forcibly displaced.^1^ Since 1975, the United States has resettled more than three million refugees, with 85,000 arriving in 2016.^2^ United States–bound refugees have three organized health encounters during resettlement: 1) required initial overseas examination, performed 2–6 months before travel to detect and treat “inadmissible” public health conditions (primarily tuberculosis [TB]). The required elements of this examination include medical history (including history of mental health or substance abuse issues), complete physical examination, evaluation for TB either by tuberculin skin test or chest X-ray (depending on age), laboratory testing for gonorrhea and syphilis, and screening for other communicable diseases of public health concern when applicable^3^; 2) predeparture examinations for fitness-to-travel and presumptive treatment of soil-transmitted helminths (STH) and *Strongyloides*, usually conducted 3–5 days pretravel by physicians from the International Organization for Migration (IOM)^4^; and 3) voluntary domestic medical examinations, performed by U.S. state or local health departments within 90 days after arrival.^4^ The Centers for Disease Control and Prevention (CDC) provides recommendations for domestic examination, but implementation varies by state.

CDC, the U.S. Department of State, and IOM have developed supplemental overseas health programs, including vaccination and presumptive STH treatment to improve and protect public and individual migrant health.^4^ These cost-saving interventions harmonize health services for refugees originating from the same areas and reduce burden on domestic providers.^5^

In 2012, we implemented a program evaluation, assessing the feasibility and impact of overseas identification and management of selected, common medical conditions beyond the scope of the required examination. To our knowledge, this is the first large-scale prospective evaluation of medical and preventive health interventions in a migrating population.

## MATERIALS AND METHODS

During the initial overseas medical examination, we offered voluntary testing and management for anemia, hepatitis B virus (HBV) infection, and intestinal parasites to a convenience sample of U.S.-bound refugees aged ≥ 6 months living in three camps on the Thailand–Burma border. These conditions were chosen because of their known high prevalence in this region^6–8^ and the potential to improve travel fitness or prevent disease by early screening or intervention. The 6-month age cutoff was chosen because physiologic anemia can occur in younger infants.^9^ Written consent was obtained from participants ≥ 15 years of age and parents or guardians of those < 15 years old (during the overseas medical examination process, refugees ≥ 15 years old are considered adults). Blood and stool samples were collected during the three examinations previously described (Supplemental Figure 1). Initial and predeparture time points were defined by dates of first and last examinations overseas, respectively, as repeat medical examinations were required for participants who did not depart within 6 months of the initial examination. Participants with identified medical conditions underwent management and evaluation based on clinical judgment and algorithms (Supplemental Figures 2–4, Supplemental Table 1). To facilitate follow-up, results were communicated to U.S. state refugee health programs directly via secure fax. States sent available, domestic examination results to CDC.

### HBV infection (Supplemental Figure 2).

All participants were screened with Alere Determine™ rapid hepatitis B surface antigen (HBsAg) test kit. HBsAg-positive participants (indicating HBV infection^6^) were counseled and evaluated (Supplemental Figure 2). HBsAg-negative participants were offered up to three doses of hepatitis B vaccine, depending on documented vaccine history and time to departure.

### Parasite infection (Supplemental Figure 3, Supplemental Table 1).

United States–bound refugees in Thailand routinely receive presumptive treatment with albendazole and ivermectin for STH and *Strongyloides stercoralis*, 24–72 hours before departure.^7^ During this pilot, participants received presumptive STH treatment at initial medical examination to assess treatment response and were retreated at predeparture to manage possible interim re-infections. Stool and blood specimens were collected before treatment. If a pathogenic parasite non-susceptible to presumptive STH therapy was identified, appropriate treatment was provided.

Stool specimens were tested for ova and parasites (O&P) by wet-mount (trichrome unavailable) and *Strongyloides* agar culture at Mae Sot General Hospital in Mae Sot, Thailand. Domestic O&P tests were performed by state clinics using concentration methods.

Participants received complete blood counts (CBC/differential). Eosinophilia was defined as absolute eosinophil count ≥ 400 cells/µL.^8^ To provide additional parasitologic insights, serum and stool were collected to evaluate (Supplemental Figure 3):

1. IgG responses to recombinant immunodiagnostic *Strongyloides stercoralis* antigen (NIE), by multiplex bead array (MBA)^10^ on paired samples (before/after treatment). A > 40% decrease in median fluorescent intensity (MFI) was considered treatment response.^11^ The cutoff for positivity was determined using three standard deviations over the mean of 61 stool-negative samples.
2. Stool (cryopreserved) quantitative polymerase chain reaction (qPCR) for *Strongyloides*, *Ascaris*, *Giardia*, Hookworm spp., *Trichuris*, and *Cryptosporidium*, using described extraction^12^ and qPCR methods^13^ (Supplemental Table 1).

### Anemia and nutrition (Supplemental Figure 4 and Supplemental Table 2).

Participants diagnosed with anemia by CBC at initial examination received management if clinically warranted. Height, weight, and mid-upper arm circumference (MUAC) were measured at initial and predeparture examinations. Eligible participants with malnutrition were referred to camp nutrition programs (Supplemental Figure 4). Weight-for-height (WHZ), body mass index (BMIZ), and height-for-age (HAZ) Z-scores were calculated using the World Health Organization SAS macro tool.^14^ Improbable Z-score and hemoglobin (Hgb) values were excluded from analysis.

Differences between groups were compared by *t* test or Wilcoxon rank-sum test for continuous variables and χ^2^ or Fisher’s exact test for categorical variables. Differences between time points were assessed using signed-rank or paired *t* test for continuous variables and McNemar’s test for categorical variables. Factors associated with chronic HBV infection, anemia, and malnutrition were assessed by logistic regression.

This project was deemed non-research by a CDC Human Subjects Advisor; IRB review was not required.

## RESULTS

We offered enrollment to all 3,419 U.S.-bound refugees having initial medical examinations during July 9, 2012 to November 29, 2013 and enrolled 57% (2,004) (Table 1). Among participants, 42% (848) were < 18 years old and 52% (1,038) were male (Table 1). Complete or partial results were available for all participants at initial, 89% (1,794) at predeparture, and 39% (777) at domestic examinations. Median time between initial and predeparture time points was 167 days (interquartile range [IQR]: 135–326; min–max: 33–1,013), and 35 days between predeparture and domestic examinations (IQR: 27–50; min–max: 11–393).

**Table 1:** Demographics and baseline (before treatment) prevalence of certain conditions among U.S.-bound refugees participating in a pilot evaluation project, Thailand–Burma border, July 2012–November 2013

Age group (years)	All participants (*N* = 2,004)		Hepatitis B (HBsAg positive; *N* = 2,004 tested)		Anemia (*N* = 2,004 tested)		Eosinophilia (Eos ≥ 0.4 K; *N* = 2,000 tested)		Stool pathogen* on qPCR (*N* = 1,839 tested)		% Participating (of *N* = 3,528 eligible)
	*M* (col %)	*F* (col %)	*M* (row %)	*F* (row %)	*M* (row %)	*F* (row %)	*M* (row %)	*F* (row %)	*M* (row %)	*F* (row %)	
< 2	37 (4)	35 (4)	0	0	25 (68)	19 (54)	29 (81)	28 (82)	19 (51)	13 (39)	72 (46)
2–4	71 (7)	81 (8)	0	0	21 (30)	18 (22)	67 (94)	65 (82)	47 (71)	557 (70)	152 (51)
5–11	193 (19)	164 (17)	1 (0.5)	3 (2)	85 (44)	66 (40)	165 (85)	116 (71)	147 (80)	117 (80)	357 (53)
12–17	154 (15)	113 (12)	21 (14)	5 (4)	63 (41)	28 (25)	109 (80)	49 (43)	113 (81)	85 (82)	267 (54)
18–29	283 (27)	267 (13)	53 (19)	28 (10)	31 (11)	55 (21)	151 (53)	86 (32)	200 (81)	151 (63)	550 (59)
30–45	184 (18)	178 (18)	31 (17)	21 (12)	18 (10)	58 (33)	100 (54)	66 (37)	130 (78)	102 (64)	362 (62)
46–64	95 (9)	99 (10)	17 (18)	10 (10)	34 (36)	26 (26)	45 (47)	40 (40)	62 (72)	61 (64)	194 (66)
≥ 65	21 (2)	29 (3)	1 (5)	0	9 (43)	13 (45)	5 (24)	9 (31)	13 (65)	22 (76)	50 (57)
Total (row %)	1,038 (52)	966 (48)	124 (12)	67 (7)	286 (28)	283 (29)	671 (65)	459 (48)	731 (77)	606 (68)	2,004 (57)

HBsAg = hepatitis B surface antigen; qPCR = quantitative polymerase chain reaction.

* *Ancylostoma duodenale*/*ceylanicum*, Ascaris lumbricoides, *Cryptosporidium parvum*/*hominum*, *Entamoeba histolytica*, *Giardia lamblia*/*intestinalis*, *Necator americanus*, *Strongyloides stercoralis*, *Trichuris trichiura*.

### HBV infection.

All 2,004 participants were tested for HBV infection at initial examination; 10% (191) had HBV infection (HBsAg-positive) and received evaluation and counseling according to Supplemental Figure 2.^6^ Most (98%) HBsAg-positive persons were ≥ 12 years old, with a 13% prevalence (187/1,423) in this group. The youngest HBsAg-positive participant was 8 years old. In multivariable analysis, odds of infection were significantly higher among males (adjusted odds ratio [aOR]: 1.92, 95% confidence interval [CI]: 1.40, 2.64) and persons ≥ 15 years (aOR: 13.18, 95% CI: 6.70, 25.93), but not among persons with tattoos. Of 79 HBsAg-positive participants aged ≥ 30 years who had liver ultrasounds overseas, 47% (37) had abnormal findings—typically hepatomegaly or parenchymal disease. At least 30 of 75 HBsAg-positive participants for whom domestic records were available were referred for further management after arrival; follow-up information was not available for the others.

Of all 1,813 HBsAg-negative participants, 19% (343) had HBsAg-positive household members; 13% (233) had previously completed the hepatitis B vaccination series, of whom 89% (208) were children < 8 years. Of the remaining 1,576, 99% (1,564) received at least one dose of vaccine at initial or predeparture examinations.

### Parasites.

**Figure 1.**
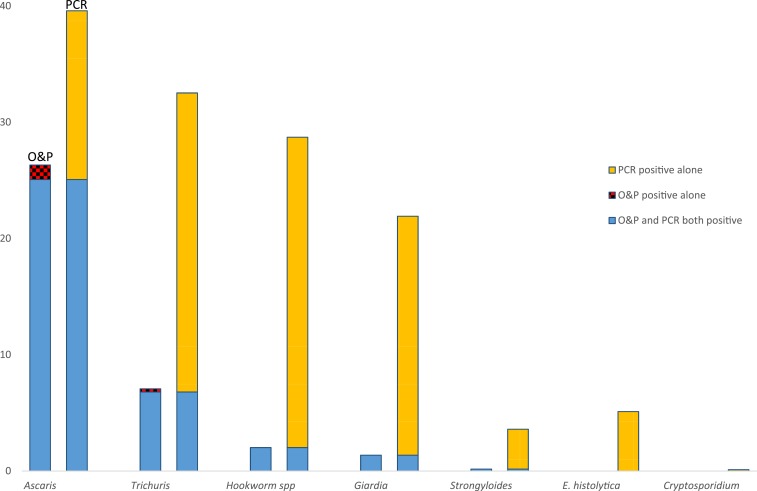
Results of intestinal parasite screening using stool ova and parasites (O&P) and quantitative polymerase chain reaction (qPCR) Among 1,839 U.S.-bound refugees participating in a pilot evaluation project, tested at initial examination using both methods, Thailand–Burma border, July 2012–November 2013. Organisms identified on O&P not tested for by qPCR were *Enterobius vermicularis* (four positive), *Taenia* spp. (four positive), and *Opisthorchis viverrini* (one positive). This figure appears in color at www.ajtmh.org.

**Figure 2.**
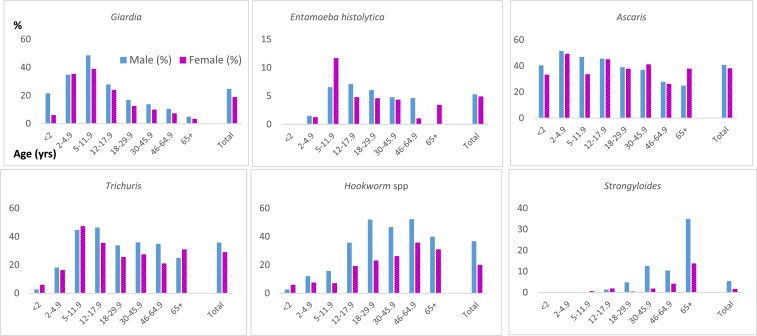
Stool quantitative polymerase chain reaction parasite prevalence at initial examination by age and sex among 2,004 U.S.-bound refugees participating in a pilot evaluation project, Thailand–Burma border, July 2012–November 2013. This figure appears in color at www.ajtmh.org.

Initial stool qPCR results—which identified more infections than stool O&P (Figure 1)—were available for 1,839 participants. At least one pathogenic organism was identified in 73% (1,337), of which 40% (743) had multiple pathogens. The most common was *Ascaris*, followed by *Trichuris* hookworm spp. (*Necator americanus*, *Ancylostoma duodenale*, *Ancylostoma ceylanicum*), and *Giardia* (Figure 2). Only 4% (66) showed *Strongyloides*. The prevalence of *Strongyloides* and hookworm increased with age, whereas prevalence of other pathogens decreased with age (Figure 2).

**Figure 3.**
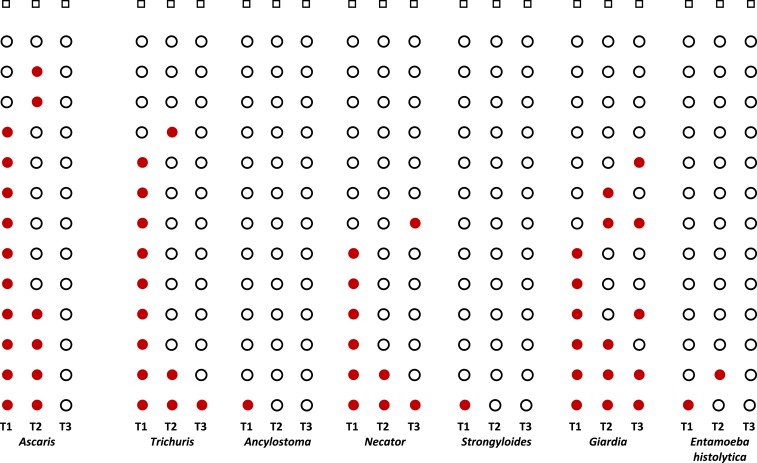
Stool quantitative polymerase chain reaction results for intestinal parasites over time for 463 U.S.-bound refugees with data at initial (T1), predeparture (T2), and domestic (T3) examinations, Thailand–Burma border, July 2012–November 2013. 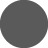 = ∼20 positive ○ = ∼20 negative □ = ∼200 negative. T1 = initial examination, T2 = pre-departure examination, and T3 = domestic examination. Each row represents the same participants over time (T1, T2, and T3 time points). This figure appears in color at www.ajtmh.org.

Among 463 participants with stool qPCR results at all time points, helminths were detected in 67% (309) at initial and 12% (57) at domestic examinations (McNemar *P* < 0.0001). Protozoan pathogens (mainly *Giardia* and *Entamoeba histolytica*) were detected in 27% (123) and 23% (107) at initial and domestic examinations, respectively (McNemar *P* = 0.1). At domestic examination, 11 helminthic and 43 protozoan infections were new. Among 1,450 participants with initial and predeparture results, there were 94 new helminthic and 193 new protozoan infections at predeparture (Figure 3).

**Figure 4.**
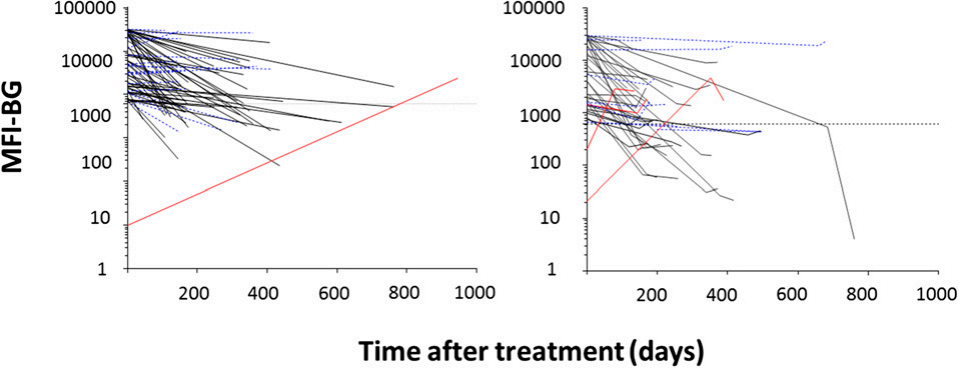
Trends in *Strongyloides* mean fluorescent intensity (MFI) over three time points for U.S.-bound refugees positive for antibodies to *Strongyloides* antigen NIE at initial examination, Thailand–Burma border, July 2012–November 2013 (*N* = 136). *y* axis shows intensity of antibody response against NIE in MFI with background subtracted (MFI-BG). *x* axis shows time in days after treatment at which point the second (predeparture) and, for right graph, third (domestic) blood collection was taken. For the right graph, the second line segment represents the time from the second to third blood collection. Each line tracks responses from a single individual who was positive for antibodies against NIE at any time point measured. Data from individuals negative at each time point are not shown. Left graph shows individuals with only two time points; right graph shows individuals with three time points. Black lines indicate individuals with at least a 40% decrease at the second or third blood collection Red lines indicate individuals negative at baseline but positive at follow-up, or an increase of greater than 40% in MFI-BG at follow-up. Dotted blue lines indicate individuals with < 40% change in MFI-BG over time. This figure appears in color at www.ajtmh.org.

*Strongyloides* stool agar was positive at initial examination in 4% (81/1,912 tested) and in 0.4% (7/1,649) at predeparture. The seropositivity of *Strongyloides* was 7% (136/1,943) at initial examination; of 118 with positive responses at any of the three examinations, 69% (82) had lower MFI at predeparture compared with initial examination, with a median decrease of 81%. No MFI changes were observed between predeparture and domestic examinations (Figure 4).

Eosinophilia was detected in 56% (1,130) of 2,000 tested at initial examination and was associated with positive stool qPCR (Fisher’s *P* < 0.0001, OR: 2.18, 95% CI: 1.77, 2.69). Eosinophilia was inversely related to age, with 84% of children < 5 years affected (Table 1). Among 521 patients with data at all three examinations, median eosinophil count decreased from 520 at initial to 380 at predeparture (signed-rank *P* < 0.001) and 310 at domestic (signed-rank *P* < 0.001) examinations. Although eosinophilia was associated with positive *Strongyloides* MBA (Fisher’s *P* < 0.001; OR: 1.94 95% CI: 1.31, 2.87) and with positive *Strongyloides* qPCR (Fisher’s *P* = 0.0077; OR: 2.1, 95% CI: 1.2, 3.6), 33% (43/132) of MBA-positive and 27% (18/66) of qPCR-positive participants were not eosinophilic.

### Anemia and nutrition.

Of all 2,004 participants, 28% (569) had anemia at initial examination; prevalence was highest among children < 2 years at 61% (44). Anemia was moderate-to-severe (Hgb < 10 g/dL) in 14% (80); 71% of these cases were in children < 5 years and women of childbearing age (18–45 years). Overseas, the most common confirmed or suspected anemia etiologies were iron deficiency (72%, 412) and thalassemia or trait (27%, 154). One asymptomatic participant was pancytopenic; these results were promptly communicated to the receiving state, leading to rapid evaluation and diagnosis of leukemia upon arrival. Etiology was not determined in 18% (104).

**Figure 5.**
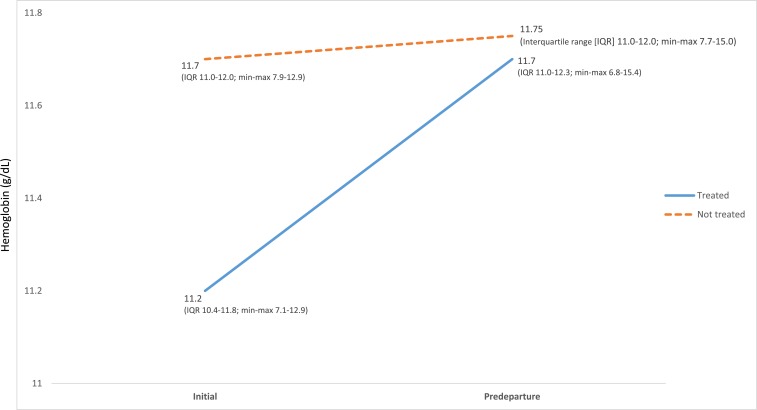
Median hemoglobin of treated (*N* = 184) vs. untreated (*N* = 182) anemic US-bound refugees at initial and predeparture examinations, Thailand–Burma border, July 2012–November 2013 (typically treated with iron, folate, B12, or multivitamin supplement, based on clinical judgment; excluding anemic patients with confirmed or suspected hemoglobinopathies/hemoglobinopathy traits who may not benefit from treatment). This figure appears in color at www.ajtmh.org.

Among 415 participants with anemia at initial examination and without thalassemia/trait, 50% (207) received overseas treatment—typically, iron or folate supplements (based on etiology/clinical judgment); one required blood transfusion. Among 366 participants with paired results, median differences between predeparture and initial Hgb were 0.4 and 0.1 g/dL for 184 treated and 182 untreated participants, respectively (rank-sum *P* < 0.001) (Figure 5).

Although anemia resolved by predeparture in 25% (142/506) of participants with results at both time points, overall initial and predeparture anemia prevalence (30%; 544/1,791) were similar; however, moderate-to-severe anemia prevalence (Hgb < 10 g/dL) was halved (14% [69/506] to 7% [36/506] at predeparture; McNemar *P* = 0.001).

Height, weight, and MUAC were measured for all participants. At initial examination, only 2% (5/224) of < 5-year-olds and 4% (26/624) of 5- to 17-year-olds had wasting, whereas 43% (97/224) of < 5-year-olds and 45% (282/624) of 5- to 17-year-olds had stunting. Of 43 pregnant women, 7% (3) had wasting. Among 197 children < 18 years with data at all three time points, mean WHZ or BMIZ increased from −0.41 ± 0.96 (95% CI: −0.55, −0.28) at initial examination to −0.23 ± 0.93 (95% CI: 0.36, −0.10) at predeparture (paired *t*, *P* < 0.001) and −0.15 ± 1.01 (95% CI: 0.29, −0.0053) at domestic examinations (paired *t*, *P* < 0.001). Mean HAZ increased from −1.86 ± 1.0 (95% CI: −2.00, −1.72) at initial examination to −1.62 ± 0.99 (95% CI: −1.76, −1.48) at domestic examination among 190 children with available data (paired *t*, *P* < 0.001).

In logistic regression models adjusted for age and sex, co-infection with helminths and protozoans on qPCR had a borderline association with anemia (ChiSq *P* = 0.0497; aOR: 1.37; 95% CI: 1.00, 1.89), whereas infection with protozoans or helminths alone did not. Among children < 18 years, co-infection with helminths and protozoans was associated with chronic malnutrition (ChiSq *P* = 0.0039; aOR: 1.81; 95% CI: 1.21, 2.71), although not with acute malnutrition. Infection with protozoans or helminths alone was not associated with malnutrition.

## DISCUSSION

This evaluation demonstrated that selected public health services, including intestinal parasite treatment, are logistically feasible and can improve health for refugees in the 2- to 6-month overseas resettlement processing window.

Our HBV screening algorithm was implemented successfully, and it identified asymptomatic patients with significant liver disease. The high (≥ 8%) prevalence of HBV infection was consistent with regional trends.^15^ Perinatal exposure, a common mode of transmission in high-prevalence settings,^16^ likely did not explain all cases, as evidenced by the higher proportion of HBV-infected males. No children < 8 years tested positive, likely due to successful camp-based vaccination programs.^17^ Early diagnosis offered an opportunity for counseling, initial management, and vaccination of contacts, and facilitated domestic follow-up.

The success and cost-benefit estimates of pre-vaccination HBsAg testing led to its incorporation into the Vaccination Program for U.S.-bound Refugees.^18–20^ This strategy may contribute to the elimination of HBV transmission in the United States^21^ and could be considered for other U.S.-bound populations, including immigrants, asylees, and other long-term visitors, such as students and workers.

The intensity and prevalence of intestinal helminths and eosinophilia decreased following presumptive STH treatment. Infection caused by *Strongyloides*, estimated to affect up to 46% of U.S.-bound refugees,^7^ is often asymptomatic and unrecognized, but can persist indefinitely if untreated. Immunosuppression, especially with corticosteroids, can result in fatal hyperinfection years after leaving an endemic area.^22,23^ Standard serologic and stool tests have poor predictive value in detecting active infection.^24^ The low prevalence of positive stool agar—thought to be a sensitive test^25^—or *Strongyloides* qPCR, may indicate that some of the 14% who were seropositive were not actively infected. Although eosinophilia was associated with positive *Strongyloides* MBA and stool qPCR, up to one-third of people testing positive were not eosinophilic, indicating that eosinophilia does not reliably predict infection.^26,27^ This assessment compares pre- and posttreatment stool and blood analyses and supports the effectiveness of mass treatment of *Strongyloides* with ivermectin.^28^ Presumptive STH and *Strongyloides* treatment has been shown to be cost-saving,^5,29^ even before the recent spike in anti-parasitic drug prices in the United States.^29^ Of note, standard-dose albendazole is minimally effective and ivermectin ineffective against protozoa (primarily, giardiasis), and significant changes in protozoan infections were neither expected nor observed. Protozoan infections are believed to have less direct public health impact in these populations,^30^ although the relationship between specific protozoans, such as *Giardia*, and malnutrition could be further explored.^31^

Identification and management of anemia overseas was associated with improved Hgb at predeparture. Severe anemia may place an individual at risk of decompensation during air travel (“fitness-to-fly”), as highlighted by the death of a refugee child while en-route to the United States in 2008 (M. Weinberg, CDC, unpublished data). However, although ancillary testing was helpful in determining anemia etiologies, it was time- and resource-intensive. In treated participants, Hgb increased significantly, but overall anemia prevalence for the group as a whole did not change. Targeted testing of high-risk groups—such as young children, women of childbearing age, and malnourished patients—may be of most benefit in this setting. Based on the predominance of iron-deficiency anemia—even among those with thalassemia/trait—an initial trial of iron or multivitamin-with-iron therapy, followed by investigation if the anemia fails to improve, may be reasonable. Caution is warranted if this approach is adopted in populations with higher malaria prevalence.^32,33^

Our results support camp survey findings of decreasing rates of acute malnutrition among children < 5 years, likely attributable to long-term camp nutritional programs, although high rates of stunting and anemia persist.^34,35^ Improvements in nutritional status among children were seen even in the short interval before departure. Further investigation may be warranted to determine whether presumptive STH treatment contributed to linear growth. Domestic results supported sustained growth improvements over time. A standard operating procedure for identification and management of moderate-to-severe acute malnutrition, based on the nutrition and anemia modules of this project, is in the process of rollout in several sites overseas.

A limitation of our evaluation was the lack of an untreated control group, due to programmatic and ethical considerations. For intestinal parasites, the possibility of re-infection between initial and predeparture examinations—which occurred in some participants (Figure 4)—was addressed by repeating presumptive STH treatment predeparture. By arrival, participants had received two courses of treatment, rather than the one predeparture course usually offered during resettlement. However, a decreased prevalence of intestinal parasites was already seen at predeparture. Also, because refugees disperse to 49 states after arrival in the United States, partial or complete domestic data were available for only 39% (777) of participants. Furthermore, laboratory testing and results available from different participating states differed based on each state’s medical examination requirements and procedures. However, all qPCR and MBA testing was performed using uniform methods at the same laboratories.

More than one-third of the U.S. states participated in this pilot. We maintained in close contact with participating states throughout, including during periodic conference calls to share findings and other updates; by direct communication with states and specific providers regarding urgent or complex cases (e.g., patients with chronic HBV infection who had evidence of liver damage) and during presentation of results and updates during annual conferences.

Several individuals, including some with life-threatening diagnoses, benefitted from earlier diagnoses and communication of health information to receiving states. However, in other instances, timely communication of overseas information was hindered by logistical challenges, including delayed documentation and electronic record transfer. It was sometimes impossible to obtain follow-up information once participants dispersed to U.S. clinics and health-care systems—highlighting the programmatic benefit of screening and delivery of simple, cost-saving interventions before arrival, and the value of optimizing health communication through the migration continuum. Continuity of care—and, therefore, access to medical data—across the resettlement process is crucial. Further exploration is needed, in collaboration with state partners, to identify best practices in documentation, patient education, and communication of health information across borders.

United States–bound refugees resettle from diverse countries and temporary asylum conditions. Health-care access and health condition prevalence vary by population. Tailoring interventions to population-specific needs, such as targeted anemia screening and selection of antiparasitic therapies, is necessary (e.g., refugees resettling from sub-Saharan Africa also receive praziquantel and artemether–lumifantrine to treat schistosomiasis and malaria, respectively^8^). Our challenge is to identify and implement those manageable, cost-effective services that would most benefit resettling refugees and provide state partners with earlier health information. In addition, clinicians who serve refugees after arrival to the United States should be aware of common conditions affecting resettling refugee populations and familiarize themselves with CDC guidance regarding screening and management of newly arrived refugees.^4^ Furthermore, they should review each refugee’s overseas medical forms and be familiar with existing predeparture interventions, such as vaccination and presumptive STH and *Strongyloides* treatment.^7,8^

Although interventions requiring longer term follow-up—such as anemia treatment and retesting, and workup and referrals for hepatitis B-positive patients—it may not be feasible in less stable settings, some of the screening we conducted relied upon, or could be accomplished using, point-of-care rapid testing. For example, pre-vaccination hepatitis B testing was conducted using a rapid test kit and counseling of positive individuals and vaccination of susceptible people can be done on the spot; although we had access to CBC in our setting, anemia screening can also be conducted using HemoCue^®^. Presumptive STH treatment with albendazole is relatively inexpensive outside of the United States and can be accomplished during a single session. Furthermore, nutritional and anthropometric assessments for children have long been used as measures of population health in unstable emergency settings^36^ and can help community and other aid agencies identify and triage those individuals needing the most acute medical intervention.

This account of our experience in implementing and assessing the impact of enhanced health screening and interventions for U.S.-bound refugees may be useful to countries resettling some of the 65 million displaced persons worldwide, as we address the shared global challenge of ensuring healthy migration for refugees and health protection for receiving communities.

## Supplementary Material

Supplemental Figure and Table.
