# Supplementary material for: Impact of Enhanced Health Interventions for United States–Bound Refugees: Evaluating Best Practices in Migration Health
Source: Am J Trop Med Hyg. 2017 Dec 18;98(3):920–8. doi: 10.4269/ajtmh.17-0725 (PMC5930906; doi:10.4269/ajtmh.17-0725)
Supplement: Supplementary file 1 [file tpmd170725.SD1.pdf]

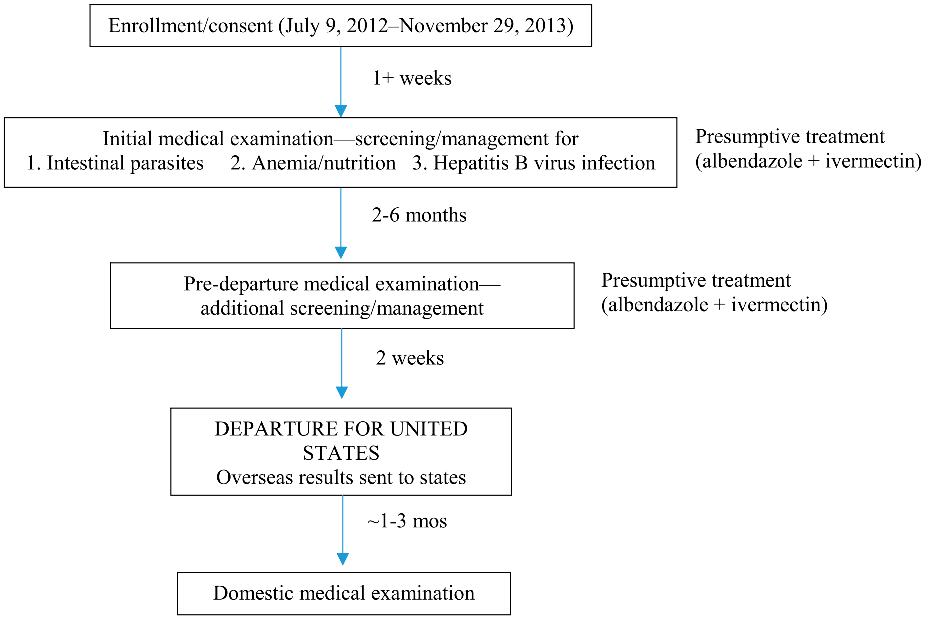

Supplemental Figure 1. Process and methods for screening and management of certain medical conditions among U.S.-bound refugees participating in a pilot evaluation project, Thailand–Burma border, July 2012–November 2013.

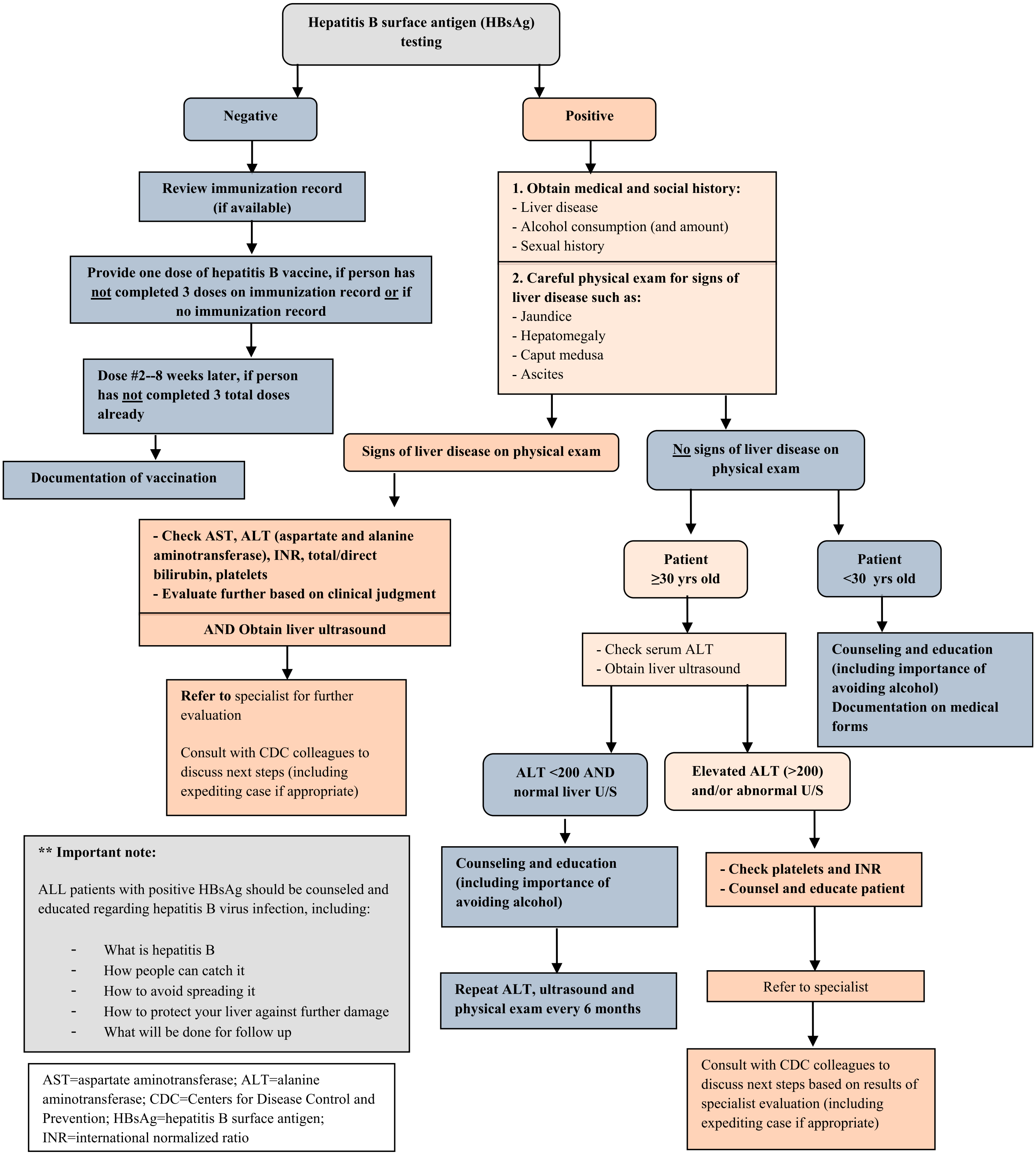

Supplemental Figure 2. Clinician guide for hepatitis B screening and management of U.S.-bound refugees participating in a pilot evaluation project, Thailand–Burma border, July 2012–November 2013.

INITIAL IOM MEDICAL ASSESSMENT

On physical exam

1. Height/weight/mid-upper-arm-circumference (MUAC) (and plot weight-for-height (WFH))

Stool tests

1. Stool ova and parasites (O&P). Ask for quantification of parasite load (1+ to 4+ or hi/med/low)
2. Agar plate for *Strongyloides*
3. Send frozen stool to National Institutes of Health (NIH) lab for qPCR

Blood tests

1. CBC/diff with eosinophils (same as for anemia testing)
2. Send frozen serum to US for multiplex bead array testing at CDC Division of Parasitic Diseases

Presumptive treatment

1. Albendazole—all participants
2. Ivermectin—all participants

Other

1. Monitor for side effects of treatment
2. *Document test results and treatment on DS form and database*
3. Treat identified pathogenic parasitic infections not covered by albendazole/ivermectin as needed

↓ **2–6 months later (in between—follow labs; treat if positive)**

PRE-DEPARTURE HEALTH SCREENING

On physical exam

1. Height/weight/MUAC (and plot WFH)

Stool tests

1. O&P. Ask for quantification of parasite load (1+ to 4+)
2. Agar plate for *Strongyloides*
3. Send frozen stool to NIH lab for qPCR

Blood tests

1. CBC/diff with eosinophils (same as for anemia testing)
2. Send frozen serum to US for multiplex bead array testing at CDC Division of Parasitic Diseases

Presumptive treatment

1. Albendazole—all participants
2. Ivermectin—all participants

Other

1. Monitor for side effects of treatment
2. Document all treatment and testing done
3. *Note: some results won't be available until after departure*

↓ **1–3 months later**

FIRST MEDICAL EXAM IN UNITED STATES

Testing per state (potentially including stool O&P, parasite serologies, stool agar, complete blood count/differential with eosinophils, anthropometric measurements)

↓ **4+ months later**

LATER MEDICAL EXAMS IN UNITED STATES

If available, as per first medical exam in the United States

SUPPLEMENTAL FIGURE 3. Clinician guide for intestinal parasite testing and presumptive treatment of U.S.-bound refugees participating in a pilot evaluation project, Thailand–Burma border, July 2012–November 2013.

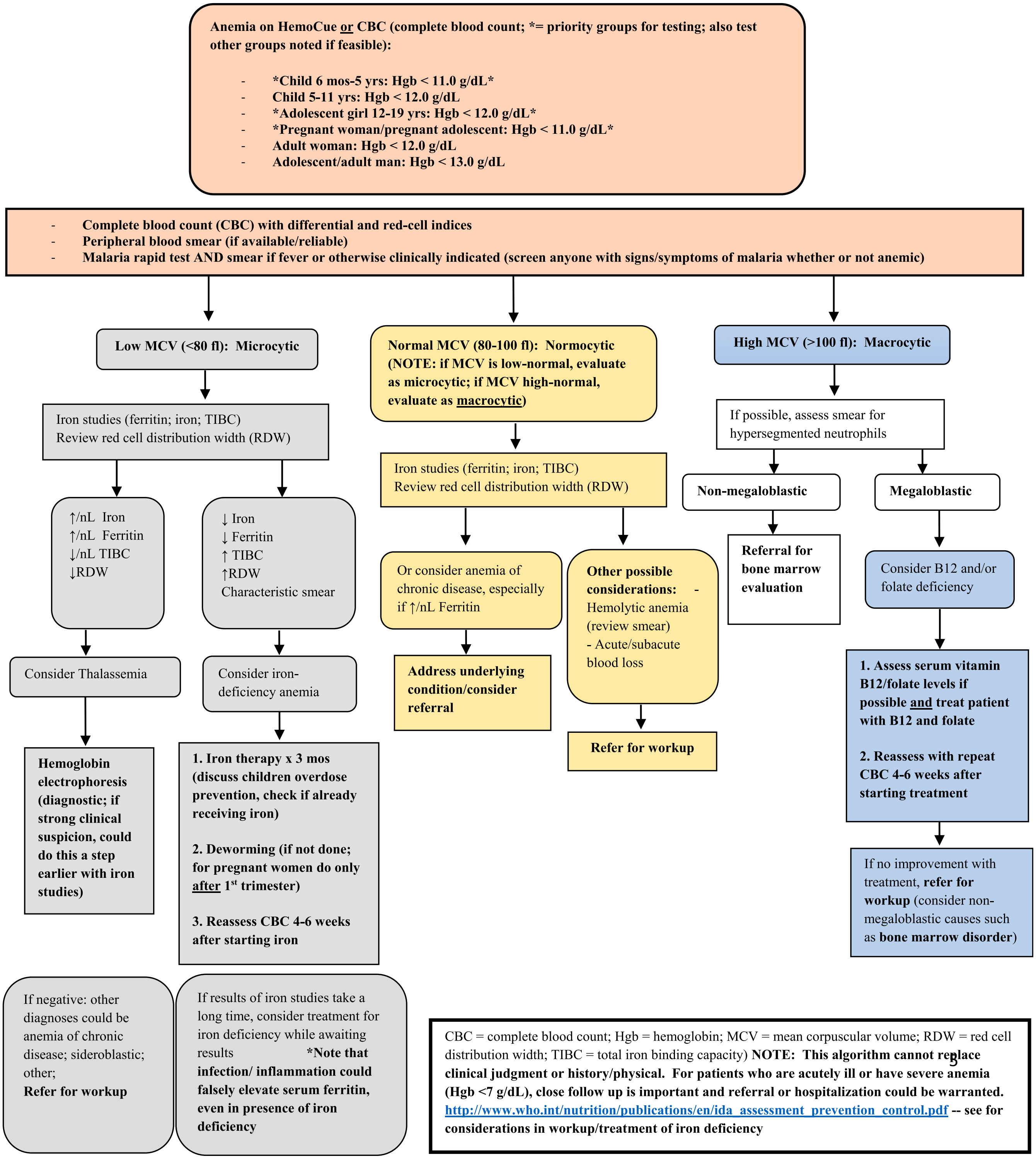

SUPPLEMENTAL FIGURE 4. Clinician guide for anemia diagnosis and management of U.S.-bound refugees participating in a pilot evaluation project, Thailand–Burma border, July 2012–November 2013.

SUPPLEMENTAL TABLE 1

Quantitative polymerase chain reaction methods to screen for intestinal parasites of U.S.-bound refugees participating in a pilot evaluation project, Thailand–Burma border, July 2012–November 2013; National Institutes of Health, Laboratory of Parasitic Diseases[37–39]

Organism	Target name	GenBank accession number	Forward primer	Reverse primer	FAM-labeled probe
Strongyloides stercoralis	Dispersed repetitive sequence	AY028262.1	CGCTCCAGAATTAGTTCCAGTT	GCAGCTTAGTCGAAAGCATAGA	5′-ACAGTCTCCAGTTCACTCCAGAAGAGT-3′
Trichuris trichiura	Dispersed repetitive sequence	HG805809.1	TTGCCTGTTGGGTGTATCTGTAA	TGCTCATCCATCCGTTGGT	5′-TAAACTTCAAAATGCCC-3′
Necator americanus	Hypothetical protein	ANCG01056619.1	CCAGAATCGCCACAAATTGTAT	GGGTTTGAGGCTTATCATAAAGAA	5′-CCCGATTTGAGCTGAATTGTCAAA-3′
Ancylostoma duodenale and *ceylanicum*	ITS2	EU344797.1	GAATGACAGCAAACTCGTTGTTG	ATACTAGCCACTGCCGAAACGT	5′-ATCGTTTACCGACTTTAG-3′
Ascaris lumbricoides	ITS1 and 5.8S	HQ721819.1	GTAATAGCAGTCGGCGGTTTCTT	GCCCAACATGCCACCTATTC	5′-TTGGCGGACAATTGCATGCGAT-3′
Cryptosporidium parvum/hominis	DNA J-like protein	XM_625506.1	AAC TTC ACG TGT GTT TGC CAA T	CCA ATC ACA GAA TCA TCA GAA TCG	5′-CATATGAAGTTATAGGGATACCAG3′
Giardia lamblia	16S rRNA	AJ293299.1	CATGCATGCCCGCTCA	AGCGGTGTCCGGTAGC	5′-AGGACAACGGTTGAC-3′
Entamoeba histolytica	18S rRNA	X75434.1	GTTTGTATTAGTACAAAATGGCCAATTC	TCGTGGCATCCTAACTCACTTAGA	5′-CAATGAATTGAGAAATACA-3′

SUPPLEMENTAL REFERENCES

37. Pilotte N, Papaiakovou M, Grant JR, Bierwert LA, Llewellyn S, McCarthy JS, Williams SA, 2016. Improved PCR-based detection of soil transmitted helminth infections using a next-generation sequencing approach to assay design. *PLoS Negl Trop Dis 10:* e0004578.
38. Basuni M, Muhi J, Othman N, Verweij JJ, Ahmad M, Miswan N, Rahumatullah A, Aziz FA, Zainudin NS, Noordin R, 2011. A pentaplex real-time polymerase chain reaction assay for detection of four species of soil-transmitted helminths. *Am J Trop Med Hyg 84:* 338–343.
39. Mejia R, Vicuña Y, Broncano N, Sandoval C, Vaca M, Chico M, Cooper PJ, Nutman TB, 2013. A novel, multi-parallel, real-time polymerase chain reaction approach for eight gastrointestinal parasites provides improved diagnostic capabilities to resource-limited at-risk populations. *Am J Trop Med Hyg 88:* 1041–1047.
40. World Health Organization, 2017. *Child Growth Standards.* Available at: http://www.who.int/childgrowth/standards/technical_report/en/. Accessed June 5, 2017.
41. United Nations Children's Fund (UNICEF), 2011. *Nutrition in Emergencies.* Available at: http://www.unicef.org/nutrition/training/index.html. Accessed June 5, 2017.
42. Centers for Disease Control and Prevention (CDC), 2002. *2000 CDC Growth Charts for the United States: Methods and Development.* Available at: https://www.cdc.gov/nchs/data/series/sr_11/sr11_246.pdf. Accessed June 5, 2017.

Supplemental Table 2

Anthropometric measurements used to define malnutrition in U.S.-bound refugees participating in a pilot evaluation project, Thailand–Burma border, July 2012–November 2013

Age group	Anthropometric measurement	Moderate acute malnutrition (wasting)	Severe acute malnutrition (wasting)	Moderate chronic malnutrition (stunting)	Severe chronic malnutrition (stunting)	Units of measurement
6–59 months	Weight-for-height Z-score	≥ –3 SD and < –2 SD	< –3 SD	–	–	kg = kilograms
5–19 years	Body mass index Z-score	≥ –3 SD and < –2 SD	< –3 SD	–	–	m = meters
6 months–19 years	Height-for-age Z-score	–	–	≥ –3 SD & < –2 SD	< –3 SD	mm = millimeters
≥ 20 years	Body mass index	< 17 to ≥ 16 kg/m^2	< 16 kg/m^2	–	–	SD = standard deviations
Pregnant women	Mid upper arm circumference	≥ 214 mm and ≤ 221 mm	< 214 mm	–	–	–
Any age	–	–	Bilateral edema	–	–	–

For children and pregnant women, based on World Health Organization and United Nations International Children's Emergency Fund standards.[40,41] For adults, based on National Center for Health Statistics standards.[42]
